# Supplementary material for: MAPK-dependent hormonal signaling plasticity contributes to overcoming Bacillus thuringiensis toxin action in an insect host
Source: Nat Commun. 2020 Jun 12;11:3003. doi: 10.1038/s41467-020-16608-8 (PMC7293236; doi:10.1038/s41467-020-16608-8)
Supplement: Supplementary file 1 — Supplementary Information [file 41467_2020_16608_MOESM1_ESM.pdf]

Supplementary Information

MAPK-dependent hormonal signaling plasticity contributes to overcoming *Bacillus thuringiensis* toxin action in an insect host

Guo et al.

This PDF file includes:

Supplementary Figures 1 to 8

Supplementary Tables 1 to 6

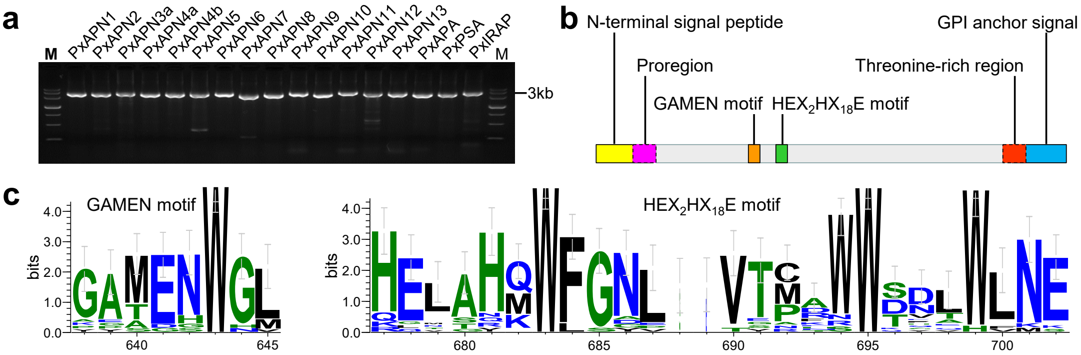

d

	PxAPN1	PxAPN2	PxAPN3a	PxAPN3b	PxAPN4a	PxAPN4b	PxAPN5	PxAPN6	PxAPN7	PxAPN8	PxAPN9	PxAPN10	PxAPN11-1	PxAPN11-2	PxAPN12	PxAPN13	PxAPA-1	PxAPA-2	PxPSA	PxIRAP
Exon 1	3(0)	482(2)	575(2)	–	4(1)	4(1)	243(0)	258(0)	470(2)	461(2)	638(2)	66(0)	183(0)	63(0)	728(2)	249(0)	195(0)	533(2)	378(0)	121(1)
Exon 2	536(2)	159(2)	168(2)	–	227(0)	224(0)	287(2)	281(2)	171(2)	159(2)	153(2)	138(0)	701(2)	701(2)	159(2)	84(0)	353(2)	136(0)	956(2)	147(1)
Exon 3	156(2)	315(2)	318(2)	–	284(2)	275(2)	159(2)	156(2)	318(2)	312(2)	294(2)	122(2)	156(2)	156(2)	294(2)	115(1)	136(0)	277(1)	190(0)	82(2)
Exon 4	318(2)	184(0)	184(0)	–	159(2)	156(2)	318(2)	155(1)	190(0)	184(0)	181(0)	195(2)	321(2)	321(2)	184(0)	97(2)	277(1)	171(1)	114(0)	122(1)
Exon 5	184(0)	154(1)	242(2)	–	149(1)	140(1)	184(0)	166(2)	242(2)	242(2)	293(2)	156(2)	184(0)	184(0)	157(1)	159(2)	171(1)	98(0)	135(0)	173(0)
Exon 6	242(2)	82(2)	193(0)	–	181(2)	181(2)	239(2)	184(0)	190(0)	190(0)	169(0)	140(1)	236(2)	236(2)	260(0)	48(2)	98(0)	135(0)	93(0)	107(2)
Exon 7	190(0)	184(0)	190(1)	–	184(0)	184(0)	190(0)	239(2)	187(1)	190(1)	178(1)	190(2)	181(0)	181(0)	202(1)	163(0)	135(0)	167(2)	150(0)	108(2)
Exon 8	190(1)	196(1)	101(0)	–	242(2)	242(2)	181(1)	190(0)	101(0)	101(0)	104(0)	151(0)	193(1)	193(1)	281(0)	213(0)	167(2)	144(2)	278(2)	73(0)
Exon 9	101(0)	140(0)	186(0)	–	190(0)	190(0)	98(0)	193(1)	189(0)	177(0)	195(0)	212(2)	275(0)	275(0)	200(2)	104(2)	144(2)	86(1)	220(0)	153(0)
Exon 10	180(0)	135(0)	200(2)	–	181(1)	181(1)	180(0)	98(0)	173(2)	185(2)	194(2)	184(0)	185(2)	185(2)	168(2)	106(0)	86(1)	131(0)	123(0)	149(2)
Exon 11	182(2)	181(1)	168(2)	–	98(0)	98(0)	185(2)	177(0)	123(2)	168(2)	177(2)	202(1)	168(2)	168(2)	144(2)	134(2)	131(0)	106(1)	213(0)	80(1)
Exon 12	315(2)	175(2)	147(2)	–	180(0)	180(0)	168(2)	188(2)	100(0)	144(2)	147(2)	275(0)	214(0)	214(0)	79(0)	196(0)	106(1)	95(0)		113(0)
Exon 13	70(0)	147(2)	76(0)	–	188(2)	188(2)	141(2)	168(2)	111(0)	70(0)	79(0)	200(2)	148(1)	148(1)	144(0)	208(1)	95(0)	77(2)		146(2)
Exon 14	300(0)	79(0)	327(0)	–	168(2)	168(2)	76(0)	144(2)		204(0)	213(0)	315(2)	221(0)	221(0)		163(2)	77(2)	158(1)		94(0)
Exon 15		228(0)			144(2)	144(2)	204(0)	79(0)				259(0)				115(0)	158(1)	130(2)		86(2)
Exon 16					79(0)	79(0)		204(0)								102(0)	130(2)	223(0)		150(2)
Exon 17					216(0)	216(0)										110(2)	223(0)	153(0)		104(1)
Exon 18																168(2)	153(0)			211(2)
Exon 19																144(2)				77(1)
Exon 20																82(0)				98(0)
Exon 21																146(2)				139(1)
Exon 22																118(0)				166(2)
Exon 23																				127(0)
Exon 24																				158(2)
Exon 25																				87(2)
Exon 26																				112(0)
Exon 27																				135(0)

e

Proteins	PxAPN1	PxAPN2	PxAPN3a	PxAPN3b	PxAPN4a	PxAPN4b	PxAPN5	PxAPN6	PxAPN7	PxAPN8	PxAPN9	PxAPN10	PxAPN11-1	PxAPN11-2	PxAPN12	PxAPN13	PxAPA-1	PxAPA-2	PxPSA	PxIRAP
PxAPN1	100%																			
PxAPN2	26%	100%																		
PxAPN3a	40%	27%	100%																	
PxAPN3b	42%	23%	52%	100%																
PxAPN4a	33%	27%	29%	29%	100%															
PxAPN4b	33%	26%	28%	29%	63%	100%														
PxAPN5	32%	26%	28%	28%	39%	36%	100%													
PxAPN6	28%	24%	26%	27%	35%	35%	39%	100%												
PxAPN7	18%	15%	15%	15%	15%	15%	18%	16%	100%											
PxAPN8	33%	27%	30%	30%	33%	31%	31%	27%	18%	100%										
PxAPN9	18%	15%	16%	16%	17%	17%	17%	17%	13%	17%	100%									
PxAPN10	19%	18%	17%	17%	18%	18%	18%	19%	14%	18%	14%	100%								
PxAPN11-1	21%	21%	21%	20%	18%	19%	18%	20%	12%	20%	14%	20%	100%							
PxAPN11-2	22%	22%	22%	21%	19%	19%	19%	21%	13%	21%	14%	20%	95%	100%						
PxAPN12	25%	27%	25%	24%	24%	25%	22%	22%	14%	27%	17%	21%	26%	27%	100%					
PxAPN13	24%	25%	22%	23%	22%	22%	22%	23%	13%	24%	14%	21%	24%	25%	29%	100%				
PxAPA-1	20%	22%	22%	19%	20%	19%	19%	21%	14%	21%	13%	19%	22%	23%	26%	23%	100%			
PxAPA-2	20%	22%	21%	20%	21%	20%	19%	21%	14%	22%	12%	18%	22%	23%	26%	24%	85%	100%		
PxPSA	22%	23%	22%	21%	23%	23%	20%	21%	14%	21%	16%	18%	22%	23%	27%	24%	30%	30%	100%	
PxIRAP	12%	11%	12%	12%	12%	10%	11%	12%	9%	10%	8%	10%	11%	11%	11%	12%	10%	10%	14%	100%

Sequence similarity

<10%	>10%	>20%	>30%	>40%	>50%	>80%	100%

Supplementary Figure 1. Genome-wide cloning and identification of PxAPN and other M1 aminopeptidase genes in *P. xylostella*. **a** Full-length cDNA clones of all *P. xylostella* PxAPN and other M1 aminopeptidase genes. **b** Schematic representation of a typical lepidopteran APN protein including six identified motifs. **c** WebLogo plots highlight amino acid conservation in the GAMEN and HEX$_2$HX$_{18}$E motifs in all of the 18 *P. xylostella* APN proteins. **d** Genomic and cDNA structure of PxAPN and

other M1 aminopeptidase genes in *P. xylostella*. Colors represent specific duplication modules during genome evolution as determined by both exon size (bp) and intron phase (in brackets). The duplication modules of *PxAPN3b* were presumed to be the same as its paralogous gene *PxAPN3a*. **e** Pairwise comparison of amino acid sequence identities among all the identified PxAPN and other M1 aminopeptidases of *P. xylostella*. Values in each rectangle represent percent similarity. Percentage similarity for each comparison is color-coded according to the gradient at the bottom. Image in (**a**) is a representative gel of three independent experiments that showed similar results. Source data are provided as a Source Data file.

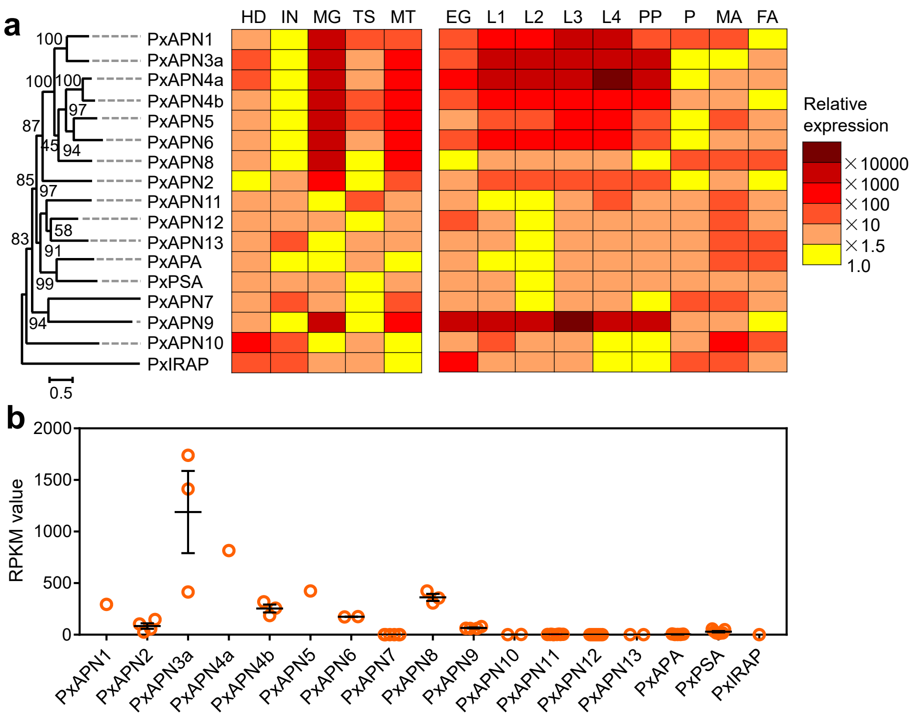

Supplementary Figure 2. Spatio-temporal expression patterns of all the PxAPN and other M1 aminopeptidase genes. a The constitutive transcription profiles of PxAPN and other M1 aminopeptidase genes in different tissues and developmental stages of DBM1Ac-S as determined by qPCR analysis. For different tissues (HD, head; IN, integument; MG, midgut; TS, testis; MT, Malpighian tubules) and developmental stages (EG, eggs; L1-4, first- to fourth-instar larvae; PP, prepupae; P, pupae; MA and FA, male and female adults). The colors of each rectangle represent the relative transcription level of each gene expressed as mean fold change relative to the counterpart with the lowest expression. Genes are organized according to their phylogenetic tree constructed by maximum likelihood method based on the optimized LG + G + I model at 690 aligned amino acid positions. **b** The absolute expression levels of PxAPN and other M1 aminopeptidase genes in midgut tissues of third-instar DBM1Ac-S larvae as determined by analyzing our previous *P. xylostella* midgut transcriptome[1] and RNA-Seq[2] libraries. Source data are provided as a Source Data file.

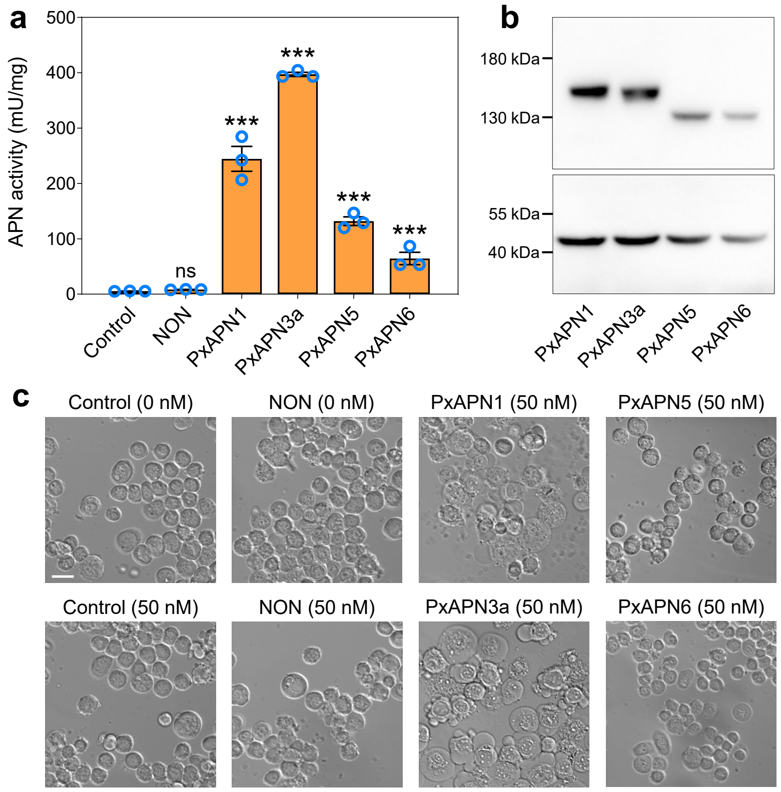

Supplementary Figure 3. Heterologous expression of PxAPN proteins and cytotoxicity observations of PxAPN-expressing Sf9 cells to Cry1Ac toxin. a Specific APN activity was measured in non-transfected wild type cells (Control), empty vector-expressing cells (NON) and PxAPN-expressing Sf9 cells. Data are presented as mean values ± SEM, n = 3 biologically independent samples, $^{***}p < 0.001$, ns, not significant, one-way ANOVA with Holm-Sidak's test was used for comparison. **b** Detection of the ectopic expression of PxAPN-GFP fusion proteins in Sf9 cells by Western blots with a mouse anti-GFP antibody. The upper detected protein bands are PxAPN proteins fused with GFP protein, and the lower protein bands are *P. xylostella* β-actin as a loading control. **c** Morphological changes of Sf9 cells following exposure to 50 nM Cry1Ac toxin. Cells were observed and representative regions were shown at 24 h after incubation with toxin. Scale bar is 20 µm. Images in (**b**, **c**) are representative blots and micrographs of three independent experiments that showed similar results, respectively. Source data are provided as a Source Data file.

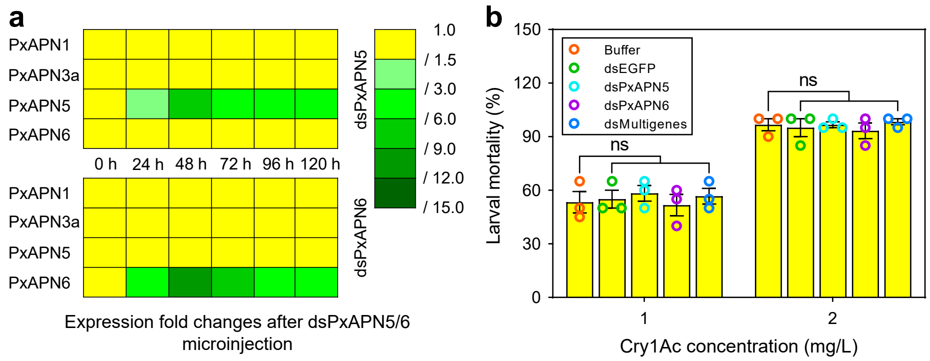

Supplementary Figure 4. Silencing of *PxAPN5* and *PxAPN6* expression and its effect on *P. xylostella* susceptibility to Cry1Ac. a RNAi-mediated silencing of *PxAPN5* or *PxAPN6* is expressed relative to transcript level for each gene at time 0 h, and are displayed as expression fold changes and color-coded according to the gradient. **b** Susceptibility to Cry1Ac protoxin in DBM1Ac-S larvae injected with buffer or dsEGFP, dsPxAPN5, dsPxAPN6 or a multiple gene mixture (dsPxAPN5 and dsPxAPN6). Data are presented as mean values (**a**) and mean values ± SEM (**b**), $n = 3$ biologically independent samples, ns, not significant, one-way ANOVA with Holm-Sidak's test was used for comparison. Source data are provided as a Source Data file.

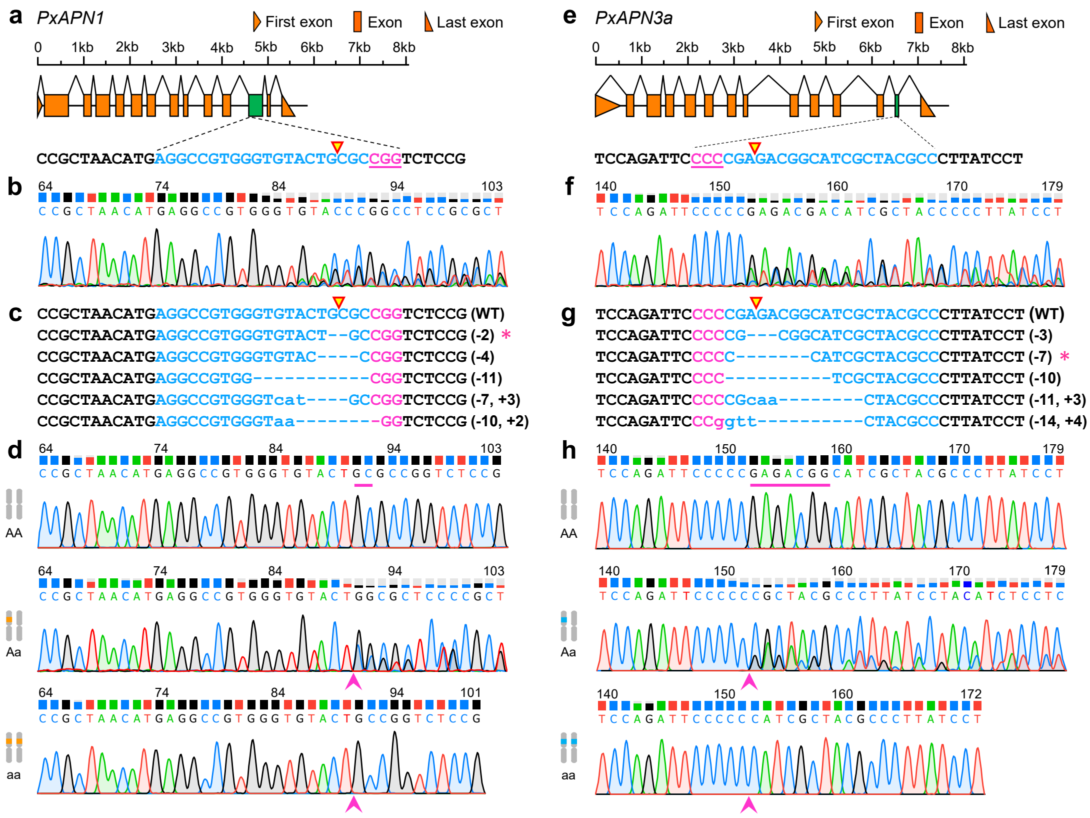

Supplementary Figure 5. CRISPR/Cas9-induced mutations of both *PxAPN1* and *PxAPN3a* genes in *P. xylostella*. **a, e** The designed sgRNA-targeted sites and sequences in exon 3 (green boxes) of both *PxAPN1* (**a**) and *PxAPN3a* (**e**). The genomic structures of both genes are drawn to scale. Exons are shown as boxes and triangles, and the spaces between two exons indicate the introns. The sgRNA target sequences of both genes are highlighted in blue, the PAM sequences are marked in pink, and the cleavage site is indicated with a purple-edged yellow inverted triangle. **b, f** Representative direct sequencing chromatograms of the pooled PCR products from mutated G0 individuals of both *PxAPN1* (**b**) and *PxAPN3a* (**f**) with a stretch of typical multiple peaks. **c, g** Various types of indel mutations flanking the CRISPR target sites of *PxAPN1* (**c**) and *PxAPN3a* (**g**) exhibited by G1 larvae, as identified by TA cloning and sequencing of the individual PCR products. Among these different mutant genotypes, the deleted bases are shown as dashes, and the inserted bases are indicated with lowercase letters. The numbers of inserted or deleted bases are displayed at the right of each allele (plus sign, insertion; minus sign, deletion). Asterisks denote the selected monoallelic mutants with enough individuals used for further sib-crossing to

generate G2 strains. **d, h** Representative chromatograms of direct sequencing of the PCR products derived from wild types (upper graph), mutant heterozygotes (middle graph) and mutant homozygotes (lower graph) of both *PxAPN1* (**d**) and *PxAPN3a* (**h**) in G2 individuals. The locations of CRISPR/Cas9-induced 2-bp deletion (GC) within exon 12 of *PxAPN1* and 7-bp deletion (GAGACGG) within exon 13 of *PxAPN3a* are marked by pink lines and arrows.

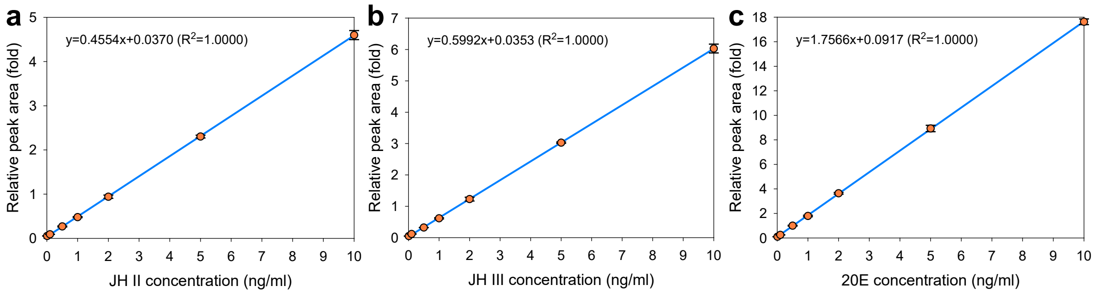

Supplementary Figure 6. The JH and 20E calibration curves. Calibration curves of JH II (**a**), JH III (**b**) and 20E (**c**). Data used for the linear regression analyses are presented as mean values ± SEM, n = 3 biologically independent samples. Source data are provided as a Source Data file.

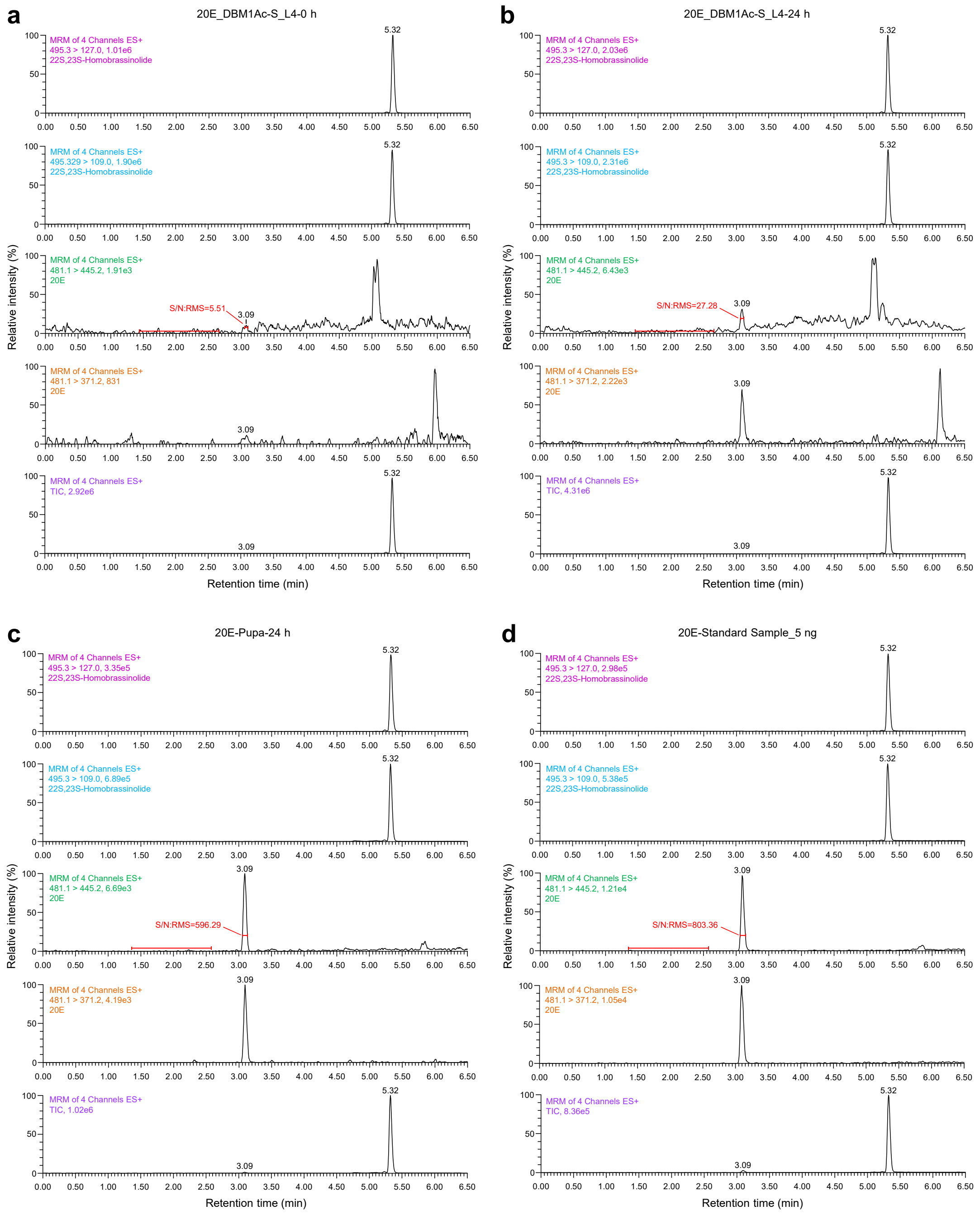

Supplementary Figure 7. Representative UPLC-MS/MS MRM chromatograms of 20E in different developmental stages. a-c Representative UPLC-MS/MS MRM chromatograms of 20E in the larval molting stage (**a**), feeding intermolt stage (**b**) and pupal stage (**c**) of DBM1Ac-S strain. **d** The UPLC-MS/MS MRM chromatogram of the standard sample of 20E (5 ng) with the internal control 22S, 23S-homobrassinolide (50 ng). MRM transitions: 20E, 481.1 > 445.2, 481.1 > 371.2; 22S, 23S-homobrassinolide, 495.3 > 127.0; 495.3 > 109.0 (also listed in Supplementary Table 5).

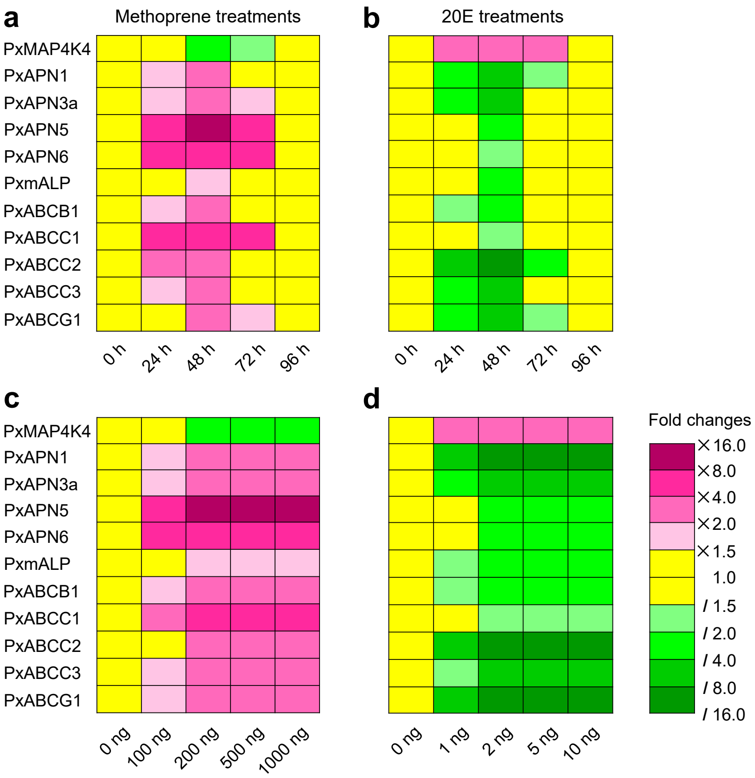

Supplementary Figure 8. Determination of optimum concentration and time post-injection of methoprene and 20E for subsequent gene expression detection. **a**, **b** Effects of 200 ng of methoprene (**a**) and 10 ng of 20E (**b**) microinjection on gene expression in DBM1Ac-S larval midgut tissues, detected at different times post-injection by qPCR. The gene expression level at 0 h was assigned a value of 1 for comparison, other levels are displayed as expression fold changes and color-coded according to the gradient. **c**, **d** Quantification of gene expression levels at 48 h post-injection with different methoprene (**c**) and 20E (**d**) concentrations by qPCR. Data are presented as mean values, n = 3 biologically independent samples, one-way ANOVA with Holm-Sidak's test was used for comparison. Source data are provided as a Source Data file.

Supplementary Table 1. Genome-wide cloning and characterization of APN and other M1 aminopeptidases in *P. xylostella*

Genes*	Gene ID[§]	Scaffold locus (Strand)[¶]	CDS (bp)	Exons	Size (aa)	pI/Mw (kDa)	Signal peptide	Glycosylation		Motifs		C-terminal GPI anchor	N-terminal TM helix
								N-Glc	O-Glc	GAMEN	HEX$_2$HX$_{18}$E		
PxAPN1	Px003754-1	**16:**2200951-2210767 (+)	2967	14	988	5.08/110.99	Yes	6	17	Yes	Yes	Yes	No
	Px008277	**296:**179000-195604 (+)											
PxAPN2	Px001708	**124:**51145-66971 (-)	2841	15	946	4.92/106.54	Yes	4	2	Yes	Yes	Yes	No
PxAPN3a	Px003753	**16:**2192000-2199719 (+)	3075	14	1024	4.74/114.97	Yes	2	8	Yes	Yes	Yes	No
	Px008276	**296:**171440-179000 (+)											
PxAPN3b[†]	N/A	N/A	2829	14	942	5.55/106.88	Yes	3	17	Yes	Yes	Yes	No
PxAPN4a	Px003754-2	**16:**2211600-2220341 (+)	2874	17	957	5.45/108.49	Yes	5	3	Yes	Yes	Yes	No
	Px008278	**296:**197537-205178 (+)											
PxAPN4b	Px003755-1	**16:**2234304-2247540 (+)	2850	17	949	5.55/107.59	Yes	4	2	Yes	Yes	Yes	No
PxAPN5	Px001705-1	**124:**2751-12464 (+)	2853	15	950	5.24/107.40	Yes	3	5	No	Yes	Yes	No
PxAPN6[‡]	Px003755-2	**16:**2248029-2252157 (+)	2880	16	959	5.15/109.61	Yes	2	5	No	Yes	Yes	No
PxAPN7	Px001706	**124:**22988-33465 (+)	2565	13	854	7.02/99.92	Yes	5	3	No	No	Yes	No
PxAPN8	Px001705-2	**124:**13280-18825 (+)	2787	14	928	5.34/103.68	Yes	3	6	Yes	Yes	Yes	No
PxAPN9	Px001707	**124:**34744-49676 (-)	3015	14	1004	4.85/113.88	Yes	3	10	No	No	Yes	No
PxAPN10	Px001711	**124:**183805-200299 (-)	2805	15	934	6.38/107.05	Yes	6	3	No	No	No	No
PxAPN11-1	Px001712	**124:**212278-221527 (+)	3366	14	1121	5.55/126.57	No	17	41	Yes	Yes	No	Yes
PxAPN11-2	Px001712	**124:**213000-221527 (+)	3246	14	1081	5.50/121.93	Yes	17	40	Yes	Yes	No	No
PxAPN12	Px003752	**16:**2172375-2188053 (+)	3000	13	999	6.43/113.40	No	10	9	Yes	Yes	No	Yes
	Px008275	**296:**129236-167825 (+)											
PxAPN13	Px005106	**19:**1272641-1296465 (-)	3024	22	1007	5.74/112.23	Yes	5	9	Yes	Yes	No	No
	Px002777	**1413:**1-5984 (-)											
PxAPA-1	Px002131+33	**131:**153617-174144 (+)	2835	18	944	8.09/108.15	No	7	3	No	Yes	No	Yes
	Px015468	**718:**43000-66645 (-)											

PxAPA-2	Px002133	**131:**162643-174144 (+)	2820	17	939	6.63/108.29	Yes	4	7	No	Yes	No	No
	Px015468	**718:**43000-64978 (-)											
PxPSA	Px001901	**128:**465492-488567 (+)	2850	11	949	6.14/107.34	No	6	7	Yes	Yes	No	No
PxIRAP	Px005721	**205:**311140-343558 (-)	3318	27	1105	6.23/121.40	No	10	26	No	No	No	Yes
	Px005938	**210:**450-31494 (-)											
	Px010493	**387:**88736-92500 (-)											
	Px015202	**699:**50517-89216 (-)											

*Based on currently available transcriptome and genome databases of *P. xylostella*, we *in silico* identified and corrected 18 aminopeptidases containing the characteristic peptidase_M1 domain (M1 aminopeptidases) including 15 aminopeptidases N (APN, membrane alanyl aminopeptidase, EC 3.4.11.2), one aminopeptidase A (APA, glutamyl aminopeptidase, EC 3.4.11.7), one puromycin-sensitive aminopeptidase (PSA, cytosol alanyl aminopeptidase, EC 3.4.11.14) and one insulin-regulated aminopeptidase (IRAP, leucyl-cystinyl aminopeptidase, EC 3.4.11.3). Both *PxAPN11* and *PxAPA* genes have two isoforms, only the first exon of *PxAPN11-1* is different from *PxAPN11-2* and the first two exons of *PxAPA-1* gene are different from exon 1 of *PxAPA-2*. The corrected full-length cDNA sequences of all these M1 aminopeptidase genes have been deposited in GenBank (accession nos. MG873047–MG873063 and MH213067–MH213068).

†Analysis of the previously identified *PxAPN3b* gene in the table is based on its partial protein sequence lacking the N-terminus (accession no. AF109692). We failed to clone the full cDNA sequence of this gene in this study, and it also can't be found in all the currently available *P. xylostella* transcriptome or genome databases, we therefore speculate that it may be a strain-specific *APN3* gene probably derived from a recent gene duplication event.

‡The exon information for *PxAPN6* gene is not complete in the genome database, so pairs of specific PCR primers were designed to obtain the complete exon information using a gDNA sample from DBM1Ac-S strain as a template (listed in Supplementary Table 6).

§Gene ID represents the gene accession number in the Diamondback Moth Genome Database (DBM-DB: http://59.79.254.1/DBM/).

¶Some M1 aminopeptidase genes may have different scaffold locus information, our analyses revealed that this is most likely to be due to uncorrected assembly and sequence redundancy in the *P. xylostella* genome. For example, our refined sequence alignment and genome scaffold analyses revealed that the small scaffold 296 is actually a part of the large scaffold 16 and that the four tandem-repeat APN genes in scaffold 296 have extremely high sequence similarity to the corresponding APN genes in scaffold 16 (the genomic sequence quality of scaffold 16 is much higher than that of scaffold 296).

Supplementary Table 2. List of the currently available lepidopteran APN and other M1 aminopeptidase genes

Name[†]	Species	Family	Source	Gene ID[‡]	Size (aa)	Sequence quality	Used in tree[§]
AjAPN1a	*Achaea janata*	Noctuidae	GenBank	ABE02186	1004	Complete	Yes
AjAPN1b	*Achaea janata*	Noctuidae	GenBank	AHA90590	997	Complete	Yes
AjAPN1c	*Achaea janata*	Noctuidae	GenBank	AHA90591	993	Complete	Yes
AjAPN2	*Achaea janata*	Noctuidae	GenBank	ASU92546	944	Complete	Yes
AjAPN3	*Achaea janata*	Noctuidae	GenBank	ASU92545	1017	Complete	Yes
AjAPN4	*Achaea janata*	Noctuidae	GenBank	ABH07377	949	Complete	Yes
AjAPN6	*Achaea janata*	Noctuidae	GenBank	ASU92547	955	Complete	Yes
AjAPN9	*Achaea janata*	Noctuidae	GenBank	ASU92549	984	Complete	Yes
AjAPA	*Achaea janata*	Noctuidae	GenBank	ASU92548	759	Partial, lacks N-terminus	No
AtAPN1	*Amyelois transitella*	Pyralidae	GenBank	XP_013184597(1)	1012	Complete, *in silico* corrected	Yes
AtAPN2	*Amyelois transitella*	Pyralidae	GenBank	XP_013184640	941	Complete	Yes
AtAPN3	*Amyelois transitella*	Pyralidae	GenBank	XP_013184605	1014	Complete	Yes
AtAPN4	*Amyelois transitella*	Pyralidae	GenBank	XP_013184597(2)	962	Complete, *in silico* corrected	Yes
AtAPN5	*Amyelois transitella*	Pyralidae	GenBank	XP_013184609	949	Complete	Yes
AtAPN6	*Amyelois transitella*	Pyralidae	GenBank	XP_013184608	956	Complete	Yes
AtAPN7	*Amyelois transitella*	Pyralidae	GenBank	XP_013184651	852	Complete	Yes
AtAPN8	*Amyelois transitella*	Pyralidae	GenBank	XP_013184610	922	Complete	Yes
AtAPN9	*Amyelois transitella*	Pyralidae	GenBank	XP_013184637	972	Complete	Yes
AtAPN10	*Amyelois transitella*	Pyralidae	GenBank	XP_013184616	936	Complete	Yes
AtAPN11	*Amyelois transitella*	Pyralidae	GenBank	XP_013184635	1139	Complete	Yes
AtAPN12	*Amyelois transitella*	Pyralidae	GenBank	XP_013184606	999	Complete	Yes
AtAPN13	*Amyelois transitella*	Pyralidae	GenBank	XP_013197300	931	Complete	Yes
AtAPA	*Amyelois transitella*	Pyralidae	GenBank	XP_013192753	1131	Complete	Yes
AtPSA	*Amyelois transitella*	Pyralidae	GenBank	XP_013190703	945	Complete	Yes

AtIRAP	*Amyelois transitella*	Pyralidae	GenBank	XP_013193451	1102	Complete	Yes
BmAPN1	*Bombyx mori*	Bombycidae	GenBank	AAC33301	986	Complete	Yes
BmAPN2	*Bombyx mori*	Bombycidae	GenBank	BAA32140	948	Complete	Yes
BmAPN3	*Bombyx mori*	Bombycidae	GenBank	AAL83943	1005	Complete	Yes
BmAPN4	*Bombyx mori*	Bombycidae	GenBank	XP_012552709	949	Complete	Yes
BmAPN5	*Bombyx mori*	Bombycidae	GenBank	AFK85018	945	Complete	Yes
BmAPN6	*Bombyx mori*	Bombycidae	SilkDB	BGIBMGA008061	891	Complete	Yes
BmAPN7	*Bombyx mori*	Bombycidae	GenBank	NP_001269149	855	Complete	Yes
BmAPN8	*Bombyx mori*	Bombycidae	GenBank	NP_001266323	930	Complete	Yes
BmAPN9	*Bombyx mori*	Bombycidae	GenBank	NP_001266329	987	Complete	Yes
BmAPN10	*Bombyx mori*	Bombycidae	GenBank	NP_001269273	941	Complete	Yes
BmAPN11	*Bombyx mori*	Bombycidae	GenBank	XP_012552714	1095	Complete	Yes
BmAPN12	*Bombyx mori*	Bombycidae	GenBank	XP_004922953	1024	Complete	Yes
BmAPN13	*Bombyx mori*	Bombycidae	GenBank	NP_001296554	936	Complete	Yes
BmAPA	*Bombyx mori*	Bombycidae	GenBank	NP_001274771	917	Complete	Yes
BmPSA	*Bombyx mori*	Bombycidae	GenBank	NP_001268818	944	Complete	Yes
BmIRAP	*Bombyx mori*	Bombycidae	GenBank	XP_021205416	1078	Complete	Yes
CmAPN3a	*Cnaphalocrocis medinalis*	Crambidae	GenBank	ADZ05466	1014	Complete	Yes
CmAPN3b	*Cnaphalocrocis medinalis*	Crambidae	GenBank	AHZ08543	1014	Complete	Yes
CmAPN4	*Cnaphalocrocis medinalis*	Crambidae	GenBank	ADZ05468	953	Complete	Yes
CmAPN5	*Cnaphalocrocis medinalis*	Crambidae	GenBank	ADZ05467	958	Complete	Yes
CsAPN1	*Chilo suppressalis*	Crambidae	GenBank	ABC69855	1075	Complete	Yes

CsAPN3a	*Chilo suppressalis*	Crambidae	GenBank	AFU51580	1002	Complete	Yes
CsAPN3b	*Chilo suppressalis*	Crambidae	GenBank	AEB96253	1013	Complete	Yes
CsAPN4	*Chilo suppressalis*	Crambidae	GenBank	ADZ57273	954	Complete	Yes
CsAPN5	*Chilo suppressalis*	Crambidae	GenBank	AFU51581	957	Complete	Yes
CsAPN6	*Chilo suppressalis*	Crambidae	GenBank	AGG36501	791	Partial, lacks N-terminus	No
DpAPN1	*Danaus plexippus*	Danaidae	GenBank	OWR41735	968	Complete	Yes
DpAPN2	*Danaus plexippus*	Danaidae	GenBank	OWR41740	939	Complete	Yes
DpAPN3	*Danaus plexippus*	Danaidae	GenBank	OWR41734	996	Complete	Yes
DpAPN4	*Danaus plexippus*	Danaidae	GenBank	EHJ64339	900	Complete	Yes
DpAPN5	*Danaus plexippus*	Danaidae	GenBank	EHJ64341	941	Complete	Yes
DpAPN6	*Danaus plexippus*	Danaidae	GenBank	EHJ64340	952	Complete	Yes
DpAPN7	*Danaus plexippus*	Danaidae	GenBank	EHJ64343+OWR41737(3)	848	Complete, deeply *in silico* corrected	Yes
DpAPN8	*Danaus plexippus*	Danaidae	GenBank	EHJ64342	928	Complete	Yes
DpAPN9	*Danaus plexippus*	Danaidae	GenBank	OWR41739	979	Complete	Yes
DpAPN10	*Danaus plexippus*	Danaidae	GenBank	OWR47405+OWR47404	875	Complete, *in silico* corrected	Yes
DpAPN11	*Danaus plexippus*	Danaidae	MonarchBase	DPOGS209841(1)	1039	Complete, deeply *in silico* corrected	Yes
DpAPN12	*Danaus plexippus*	Danaidae	GenBank	OWR41732	995	Complete	Yes
DpAPN13	*Danaus plexippus*	Danaidae	GenBank	EHJ69867	965	Complete	Yes
DpAPA	*Danaus plexippus*	Danaidae	MonarchBase	DPOGS208533	635	Partial, lacks N-terminus	No
DpPSA	*Danaus plexippus*	Danaidae	GenBank	OWR48461	866	Complete	Yes
DpIRAP	*Danaus plexippus*	Danaidae	GenBank	EHJ74954	684	Partial, lacks N-terminus	No
DsAPN1	*Diatraea saccharalis*	Crambidae	GenBank	ADL38968	1117	Complete	Yes
DsAPN2	*Diatraea saccharalis*	Crambidae	GenBank	ADL38969	940	Complete	Yes
DsAPN3	*Diatraea saccharalis*	Crambidae	GenBank	ADL38970	1012	Complete	Yes

EpAPN3	*Epiphyas postvittana*	Tortricidae	GenBank	AAF99701	1007	Complete	Yes
HaAPN1	*Helicoverpa armigera*	Noctuidae	GenBank	AAK85538	1014	Complete	Yes
HaAPN2	*Helicoverpa armigera*	Noctuidae	GenBank	XP_021192782	941	Complete	Yes
HaAPN3	*Helicoverpa armigera*	Noctuidae	GenBank	AAL14117	1014	Complete	Yes
HaAPN4	*Helicoverpa armigera*	Noctuidae	GenBank	AAP37950	951	Complete	Yes
HaAPN5	*Helicoverpa armigera*	Noctuidae	GenBank	AAP37951	1032	Complete	Yes
HaAPN6	*Helicoverpa armigera*	Noctuidae	GenBank	ACA35025	961	Complete	Yes
HaAPN7	*Helicoverpa armigera*	Noctuidae	GenBank	XP_021192778	863	Complete	Yes
HaAPN8	*Helicoverpa armigera*	Noctuidae	GenBank	XP_021192784	939	Complete	Yes
HaAPN9	*Helicoverpa armigera*	Noctuidae	GenBank	XP_021192786	985	Complete	Yes
HaAPN10	*Helicoverpa armigera*	Noctuidae	GenBank	XP_021197747	942	Complete	Yes
HaAPN11	*Helicoverpa armigera*	Noctuidae	GenBank	XP_021197746	1066	Complete	Yes
HaAPN12	*Helicoverpa armigera*	Noctuidae	GenBank	XP_021192750	1003	Complete	Yes
HaAPN13	*Helicoverpa armigera*	Noctuidae	GenBank	XP_021190039	974	Complete	Yes
HaAPA	*Helicoverpa armigera*	Noctuidae	GenBank	XP_021183618	937	Complete	Yes
HaPSA	*Helicoverpa armigera*	Noctuidae	GenBank	XP_021195025	948	Complete	Yes
HaIRAP	*Helicoverpa armigera*	Noctuidae	GenBank	XP_021193659+XP_021193652	1133	Complete, *in silico* corrected	Yes
HpAPN1	*Helicoverpa punctigera*	Noctuidae	GenBank	AAF37558	1011	Complete	Yes
HpAPN3	*Helicoverpa punctigera*	Noctuidae	GenBank	AAF37560	1013	Complete	Yes
HpAPN4	*Helicoverpa punctigera*	Noctuidae	GenBank	AAF37559	952	Complete	Yes
HvAPN1	*Heliothis virescens*	Noctuidae	GenBank	AAF08254	1010	Complete	Yes
HvAPN2	*Heliothis virescens*	Noctuidae	GenBank	PCG71579+PCG66278	940	Complete, deeply *in silico* corrected	Yes
HvAPN3	*Heliothis virescens*	Noctuidae	GenBank	AAC46929	1009	Complete	Yes
HvAPN4	*Heliothis virescens*	Noctuidae	GenBank	AAK58066	950	Complete	Yes

HvAPN5	*Heliothis virescens*	Noctuidae	GenBank	PCG64448	1047	Complete	Yes
HvAPN6	*Heliothis virescens*	Noctuidae	GenBank	PCG68031	963	Complete	Yes
HvAPN7	*Heliothis virescens*	Noctuidae	GenBank	PCG62786	257	Partial, lacks N and C-termini	No
HvAPN8	*Heliothis virescens*	Noctuidae	GenBank	PCG64451	936	Complete	Yes
HvAPN9	*Heliothis virescens*	Noctuidae	GenBank	PCG66277	971	Complete	Yes
HvAPN10	*Heliothis virescens*	Noctuidae	GenBank	PCG70717	937	Complete	Yes
HvAPN11	*Heliothis virescens*	Noctuidae	GenBank	PCG70716	1048	Complete	Yes
HvAPN12	*Heliothis virescens*	Noctuidae	GenBank	PCG65583	989	Complete	Yes
HvAPN13	*Heliothis virescens*	Noctuidae	GenBank	PCG74619	1405	Complete	Yes
HvAPA	*Heliothis virescens*	Noctuidae	GenBank	PCG70651	938	Complete	Yes
HvIRAP	*Heliothis virescens*	Noctuidae	GenBank	PCG79066	1050	Complete	Yes
LdAPN1	*Lymantria dispar*	Lymantriidae	GenBank	AAL26895	995	Complete	Yes
LdAPN2	*Lymantria dispar*	Lymantriidae	GenBank	AAD31184	942	Complete	Yes
LdAPN3	*Lymantria dispar*	Lymantriidae	GenBank	AAD31183	1017	Complete	Yes
LdAPN4	*Lymantria dispar*	Lymantriidae	GenBank	AAL26894	947	Complete	Yes
MbAPN3	*Mamestra brassicae*	Noctuidae	GenBank	AHW57924	1006	Complete	Yes
McAPN1	*Mamestra configurata*	Noctuidae	GenBank	AEA76302	1036	Complete	Yes
McAPN2	*Mamestra configurata*	Noctuidae	GenBank	AEA76305	373	Partial, lacks C-terminus	No
McAPN3	*Mamestra configurata*	Noctuidae	GenBank	AEA76296+AEA76297	578	Partial, lacks N and C-termini	No
McAPN4	*Mamestra configurata*	Noctuidae	GenBank	AEA76299+AEA76300+AEA76301+ACN69219	949	Complete, deeply *in silico* corrected	Yes
McAPN5	*Mamestra configurata*	Noctuidae	GenBank	AEA76295	242	Partial, lacks C-terminus	No
McAPN6	*Mamestra configurata*	Noctuidae	GenBank	AEA76303+ACN69221+AEA76304	888	Complete, deeply *in silico* corrected	Yes

MsAPN1	*Manduca sexta*	Sphingidae	GenBank	AAF07223	995	Complete	Yes
MsAPN2	*Manduca sexta*	Sphingidae	GenBank	CAA66466	942	Complete	Yes
MsAPN3a	*Manduca sexta*	Sphingidae	Manduca Base	Msex2.10158	1010	Complete	Yes
MsAPN3b	*Manduca sexta*	Sphingidae	Manduca Base	Msex2.10157	1010	Complete	Yes
MsAPN4	*Manduca sexta*	Sphingidae	GenBank	AAM18718	947	Complete	Yes
MsAPN5	*Manduca sexta*	Sphingidae	Manduca Base	Msex2.10153	947	Complete	Yes
MsAPN6	*Manduca sexta*	Sphingidae	Manduca Base	Msex2.10154	955	Complete	Yes
MsAPN7	*Manduca sexta*	Sphingidae	Manduca Base	Msex2.10151	855	Complete	Yes
MsAPN8	*Manduca sexta*	Sphingidae	Manduca Base	Msex2.10152	927	Complete	Yes
MsAPN9	*Manduca sexta*	Sphingidae	Manduca Base	Msex2.10149	988	Complete	Yes
MsAPN10	*Manduca sexta*	Sphingidae	Manduca Base	Msex2.05162	938	Complete	Yes
MsAPN11	*Manduca sexta*	Sphingidae	Manduca Base	Msex2.05163	1131	Complete	Yes
MsAPN12	*Manduca sexta*	Sphingidae	Manduca Base	Msex2.10159	988	Complete	Yes
MsAPN13	*Manduca sexta*	Sphingidae	Manduca Base	Msex2.14756	969	Complete	Yes
MsAPA	*Manduca sexta*	Sphingidae	Manduca Base	Msex2.11506	916	Complete	Yes
MsPSA	*Manduca sexta*	Sphingidae	Manduca Base	Msex2.02406	948	Complete	Yes
MsIRAP	*Manduca sexta*	Sphingidae	Manduca Base	MCOT.C11373+74.0.0.OOO2B_X	793	Partial, lacks N and C-termini	No
OfAPN1	*Ostrinia furnacalis*	Crambidae	GenBank	ACX85727	994	Complete	Yes
OfAPN2	*Ostrinia furnacalis*	Crambidae	GenBank	ACB47287	940	Complete	Yes
OfAPN3	*Ostrinia furnacalis*	Crambidae	GenBank	ABV01346	1014	Complete	Yes
OfAPN4	*Ostrinia furnacalis*	Crambidae	GenBank	ACB87202	951	Complete	Yes
OnAPN1	*Ostrinia nubilalis*	Crambidae	GenBank	ACJ64827	994	Complete	Yes
OnAPN2	*Ostrinia nubilalis*	Crambidae	GenBank	ACJ64828	940	Complete	Yes
OnAPN3a	*Ostrinia nubilalis*	Crambidae	GenBank	ADA57169	1014	Complete	Yes
OnAPN3b	*Ostrinia nubilalis*	Crambidae	GenBank	ACT35083	1017	Complete	Yes

OnAPN3c	*Ostrinia nubilalis*	Crambidae	GenBank	AEO12697	1074	Complete	Yes
OnAPN3d	*Ostrinia nubilalis*	Crambidae	GenBank	AEO12695	603	Partial, lacks N-terminus	No
OnAPN4	*Ostrinia nubilalis*	Crambidae	GenBank	ACV74256	951	Complete	Yes
OnAPN5a	*Ostrinia nubilalis*	Crambidae	GenBank	AGO57907	953	Complete	Yes
OnAPN5b	*Ostrinia nubilalis*	Crambidae	GenBank	AEO12694	959	Complete	Yes
OnAPN6	*Ostrinia nubilalis*	Crambidae	GenBank	AGO57906	953	Complete	Yes
OnAPN7	*Ostrinia nubilalis*	Crambidae	GenBank	AEO12692	842	Complete	Yes
OnAPN8	*Ostrinia nubilalis*	Crambidae	GenBank	ACV04931	925	Complete	Yes
OnPSA	*Ostrinia nubilalis*	Crambidae	GenBank	ACT35084	555	Partials, lack C-terminus	No
ObAPN1	*Operophtera brumata*	Geometridae	GenBank	KOB73138(2)	923	Partial, lacks N-terminus and intermediate segment	No
ObAPN2	*Operophtera brumata*	Geometridae	GenBank	KOB65200	549	Partial, lacks C-terminus	No
ObAPN3	*Operophtera brumata*	Geometridae	GenBank	KOB73138(1)	981	Partial, lacks C-terminus	No
ObAPN4	*Operophtera brumata*	Geometridae	GenBank	KOB70212	594	Partial, lacks N-terminus	No
ObAPN5	*Operophtera brumata*	Geometridae	GenBank	KOB70211(2)	936	Partial, lacks intermediate segment	No
ObAPN6	*Operophtera brumata*	Geometridae	GenBank	KOB70211(1)	814	Partial, lacks intermediate segment and C-terminus	No
ObAPN7	*Operophtera brumata*	Geometridae	GenBank	KOB74748	371	Partial, lacks N and C-termini	No
ObAPN8	*Operophtera brumata*	Geometridae	GenBank	KOB70213	570	Partial, lacks intermediate segment and C-terminus	No
ObAPN9	*Operophtera brumata*	Geometridae	GenBank	KOB74749	817	Partial, lacks intermediate segment	No
ObAPN10	*Operophtera brumata*	Geometridae	GenBank	KOB68372	459	Partial, lacks N and C-termini	No

ObAPN11	*Operophtera brumata*	Geometridae	GenBank	KOB79054+KOB77558	1000	Partial, lacks N and C-termini	No
ObAPN12	*Operophtera brumata*	Geometridae	GenBank	KOB73704+KOB52102+KOB73139	791	Partial, lacks N-terminus	No
ObAPN13	*Operophtera brumata*	Geometridae	GenBank	KOB70715+KOB70714	789	Partial, lacks N and C-termini	No
ObAPA	*Operophtera brumata*	Geometridae	GenBank	KOB64146	375	Partial, lacks N and C-termini	No
ObPSA	*Operophtera brumata*	Geometridae	GenBank	KOB73217	873	Partial, lacks intermediate segment	No
ObIRAP	*Operophtera brumata*	Geometridae	GenBank	KOB68844	459	Partial, lacks N and C-termini	No
PiAPN3	*Plodia interpunctella*	Pyralidae	GenBank	AAC36148	1016	Complete	Yes
PmAPN1	*Papilio machaon*	Papilionidae	GenBank	XP_014370421	992	Complete	Yes
PmAPN2	*Papilio machaon*	Papilionidae	GenBank	XP_014370554	940	Complete	Yes
PmAPN3	*Papilio machaon*	Papilionidae	GenBank	XP_014370443	1004	Complete	Yes
PmAPN4	*Papilio machaon*	Papilionidae	GenBank	XP_014370422	949	Complete	Yes
PmAPN5	*Papilio machaon*	Papilionidae	GenBank	XP_014370487	952	Complete	Yes
PmAPN6	*Papilio machaon*	Papilionidae	GenBank	XP_014370483	950	Complete	Yes
PmAPN7	*Papilio machaon*	Papilionidae	GenBank	XP_014370153	707	Complete	Yes
PmAPN8	*Papilio machaon*	Papilionidae	GenBank	XP_014370556	928	Complete	Yes
PmAPN9	*Papilio machaon*	Papilionidae	GenBank	XP_014370589	992	Complete	Yes
PmAPN10	*Papilio machaon*	Papilionidae	GenBank	XP_014370234	929	Complete	Yes
PmAPN11	*Papilio machaon*	Papilionidae	GenBank	XP_014370557	1087	Complete	Yes
PmAPN12	*Papilio machaon*	Papilionidae	GenBank	XP_014370444	1002	Complete	Yes
PmAPN13	*Papilio machaon*	Papilionidae	GenBank	XP_014365203	978	Complete	Yes

PmAPA	*Papilio machaon*	Papilionidae	GenBank	XP_014362466	820	Complete	Yes
PmPSA	*Papilio machaon*	Papilionidae	GenBank	XP_014368965	956	Complete	Yes
PmIRAP	*Papilio machaon*	Papilionidae	GenBank	XP_014357412+XP_014357425	966	Partial, lacks intermediate segment	No
PpAPN1	*Papilio polytes*	Papilionidae	GenBank	XP_013142035	986	Complete	Yes
PpAPN2	*Papilio polytes*	Papilionidae	GenBank	XP_013142058	940	Complete	Yes
PpAPN3	*Papilio polytes*	Papilionidae	GenBank	XP_013142027	1003	Complete	Yes
PpAPN4	*Papilio polytes*	Papilionidae	GenBank	XP_013142032	949	Complete	Yes
PpAPN5	*Papilio polytes*	Papilionidae	GenBank	XP_013142029	952	Complete	Yes
PpAPN6	*Papilio polytes*	Papilionidae	GenBank	XP_013142030	951	Complete	Yes
PpAPN7	*Papilio polytes*	Papilionidae	GenBank	XP_013142034	853	Complete	Yes
PpAPN8	*Papilio polytes*	Papilionidae	GenBank	XP_013142033	928	Complete	Yes
PpAPN9	*Papilio polytes*	Papilionidae	GenBank	XP_013142059	990	Complete	Yes
PpAPN10	*Papilio polytes*	Papilionidae	GenBank	XP_013142020	932	Complete	Yes
PpAPN11	*Papilio polytes*	Papilionidae	GenBank	XP_013142048	1063	Complete	Yes
PpAPN12	*Papilio polytes*	Papilionidae	GenBank	XP_013142028	1000	Complete	Yes
PpAPN13	*Papilio polytes*	Papilionidae	GenBank	XP_013141776	794	Partial, lacks N-terminus	No
PpAPA	*Papilio polytes*	Papilionidae	GenBank	XP_013142752	938	Complete	Yes
PpPSA	*Papilio polytes*	Papilionidae	GenBank	XP_013146811	959	Complete	Yes
PpIRAP	*Papilio polytes*	Papilionidae	GenBank	XP_013141756+XP_013141755	1072	Complete, *in silico* corrected	Yes
PrAPN1	*Pieris rapae*	Pieridae	GenBank	XP_022120580(2)	882	Partial, lacks C-terminus	No
PrAPN2	*Pieris rapae*	Pieridae	GenBank	XP_022125612	930	Complete	Yes
PrAPN3	*Pieris rapae*	Pieridae	GenBank	XP_022120580(1)	1015	Complete	Yes
PrAPN4	*Pieris rapae*	Pieridae	GenBank	XP_022128349	947	Complete	Yes
PrAPN5	*Pieris rapae*	Pieridae	GenBank	XP_022125615	943	Complete	Yes

PrAPN6	*Pieris rapae*	Pieridae	GenBank	XP_022125616	953	Complete	Yes
PrAPN7	*Pieris rapae*	Pieridae	GenBank	XP_022125618	849	Complete	Yes
PrAPN8	*Pieris rapae*	Pieridae	GenBank	XP_022125617	929	Complete	Yes
PrAPN9	*Pieris rapae*	Pieridae	GenBank	XP_022125613	990	Complete	Yes
PrAPN10	*Pieris rapae*	Pieridae	GenBank	XP_022127014	929	Complete	Yes
PrAPN11	*Pieris rapae*	Pieridae	GenBank	XP_022127003	1048	Complete	Yes
PrAPN12	*Pieris rapae*	Pieridae	GenBank	XP_022120582	1006	Complete	Yes
PrAPN13	*Pieris rapae*	Pieridae	GenBank	XP_022113951	962	Complete	Yes
PrAPA	*Pieris rapae*	Pieridae	GenBank	XP_022123195	923	Complete	Yes
PrPSA	*Pieris rapae*	Pieridae	GenBank	XP_022130081	955	Complete	Yes
PrIRAP	*Pieris rapae*	Pieridae	GenBank	XP_022119177	1073	Complete	Yes
PxuAPN1	*Papilio xuthus*	Papilionidae	GenBank	XP_013171122	990	Complete	Yes
PxuAPN2	*Papilio xuthus*	Papilionidae	GenBank	XP_013171724	940	Complete	Yes
PxuAPN3	*Papilio xuthus*	Papilionidae	GenBank	XP_013171863	1003	Complete	Yes
PxuAPN4	*Papilio xuthus*	Papilionidae	GenBank	XP_013171158	949	Complete	Yes
PxuAPN5	*Papilio xuthus*	Papilionidae	GenBank	KPJ03375	948	Complete	Yes
PxuAPN6	*Papilio xuthus*	Papilionidae	GenBank	XP_013171146	950	Complete	Yes
PxuAPN7	*Papilio xuthus*	Papilionidae	GenBank	XP_013172178	857	Complete	Yes
PxuAPN8	*Papilio xuthus*	Papilionidae	GenBank	XP_013171170	928	Complete	Yes
PxuAPN9	*Papilio xuthus*	Papilionidae	GenBank	XP_013171712	979	Complete	Yes
PxuAPN10	*Papilio xuthus*	Papilionidae	GenBank	XP_013172351	930	Complete	Yes
PxuAPN11	*Papilio xuthus*	Papilionidae	GenBank	XP_013171913	1093	Complete	Yes
PxuAPN12	*Papilio xuthus*	Papilionidae	GenBank	KPJ03377	1001	Complete	Yes
PxuAPN13	*Papilio xuthus*	Papilionidae	GenBank	XP_013165599	976	Complete	Yes
PxuAPA	*Papilio xuthus*	Papilionidae	GenBank	XP_013180274	915	Complete	Yes
PxuPSA	*Papilio xuthus*	Papilionidae	GenBank	XP_013172369	956	Complete	Yes

PxuIRAP	*Papilio xuthus*	Papilionidae	GenBank	KPJ00497	1092	Complete	Yes
PxyAPN1	*Plutella xylostella*	Plutellidae	GenBank	MG873047	988	Complete, cloned	Yes
PxyAPN2	*Plutella xylostella*	Plutellidae	GenBank	MG873048	946	Complete, cloned	Yes
PxyAPN3a	*Plutella xylostella*	Plutellidae	GenBank	MG873049	1024	Complete, cloned	Yes
PxyAPN3b	*Plutella xylostella*	Plutellidae	GenBank	AAF01259	942	Partial, lacks N-terminus	No
PxyAPN4a	*Plutella xylostella*	Plutellidae	GenBank	MG873050	957	Complete, cloned	Yes
PxyAPN4b	*Plutella xylostella*	Plutellidae	GenBank	MG873051	949	Complete, cloned	Yes
PxyAPN5	*Plutella xylostella*	Plutellidae	GenBank	MG873052	950	Complete, cloned	Yes
PxyAPN6	*Plutella xylostella*	Plutellidae	GenBank	MG873053	959	Complete, cloned	Yes
PxyAPN7	*Plutella xylostella*	Plutellidae	GenBank	MG873054	854	Complete, cloned	Yes
PxyAPN8	*Plutella xylostella*	Plutellidae	GenBank	MG873055	928	Complete, cloned	Yes
PxyAPN9	*Plutella xylostella*	Plutellidae	GenBank	MG873056	1004	Complete, cloned	Yes
PxyAPN10	*Plutella xylostella*	Plutellidae	GenBank	MG873057	934	Complete, cloned	Yes
PxyAPN11-1	*Plutella xylostella*	Plutellidae	GenBank	MG873058	1121	Complete, cloned	Yes
PxyAPN11-2	*Plutella xylostella*	Plutellidae	GenBank	MH213067	1081	Complete, cloned	Yes
PxyAPN12	*Plutella xylostella*	Plutellidae	GenBank	MG873059	999	Complete, cloned	Yes
PxyAPN13	*Plutella xylostella*	Plutellidae	GenBank	MG873060	1007	Complete, cloned	Yes
PxyAPA-1	*Plutella xylostella*	Plutellidae	GenBank	MH213068	944	Complete, cloned	Yes
PxyAPA-2	*Plutella xylostella*	Plutellidae	GenBank	MG873061	916	Complete, cloned	Yes
PxyPSA	*Plutella xylostella*	Plutellidae	GenBank	MG873062	949	Complete, cloned	Yes
PxyIRAP	*Plutella xylostella*	Plutellidae	GenBank	MG873063	1105	Complete, cloned	Yes
SeAPN1	*Spodoptera exigua*	Noctuidae	GenBank	AAP44964	1021	Complete	Yes
SeAPN2	*Spodoptera exigua*	Noctuidae	GenBank	AAP44965	960	Complete	Yes
SeAPN3	*Spodoptera exigua*	Noctuidae	GenBank	AAP44966	1005	Complete	Yes

SeAPN4	*Spodoptera exigua*	Noctuidae	GenBank	AAP44967	951	Complete	Yes
SeAPN5	*Spodoptera exigua*	Noctuidae	GenBank	AIK27005	980	Complete	Yes
SeAPN6	*Spodoptera exigua*	Noctuidae	GenBank	AIK27006	957	Complete	Yes
SiAPN3	*Sesamia inferens*	Noctuidae	GenBank	AEL22855	1006	Complete	Yes
SlittAPN4	*Spodoptera littoralis*	Noctuidae	GenBank	ADZ74247	952	Complete	Yes
SlituAPN1	*Spodoptera litura*	Noctuidae	GenBank	XP_022835594	1000	Complete	Yes
SlituAPN2	*Spodoptera litura*	Noctuidae	GenBank	XP_022825009	974	Complete	Yes
SlituAPN3	*Spodoptera litura*	Noctuidae	GenBank	XP_022834840	1011	Complete	Yes
SlituAPN4	*Spodoptera litura*	Noctuidae	GenBank	AAK69605	952	Complete	Yes
SlituAPN5	*Spodoptera litura*	Noctuidae	GenBank	XP_022825006	1005	Complete	Yes
SlituAPN6	*Spodoptera litura*	Noctuidae	GenBank	XP_022825010	953	Complete	Yes
SlituAPN7	*Spodoptera litura*	Noctuidae	GenBank	XP_022825013	862	Complete	Yes
SlituAPN8	*Spodoptera litura*	Noctuidae	GenBank	XP_022825012	936	Complete	Yes
SlituAPN9	*Spodoptera litura*	Noctuidae	GenBank	XP_022825007	978	Complete	Yes
SlituAPN10	*Spodoptera litura*	Noctuidae	GenBank	XP_022825011	938	Complete	Yes
SlituAPN11	*Spodoptera litura*	Noctuidae	GenBank	XP_022825002	1099	Complete	Yes
SlituAPN12	*Spodoptera litura*	Noctuidae	GenBank	XP_022834841	1005	Complete	Yes
SlituAPN13	*Spodoptera litura*	Noctuidae	GenBank	XP_022827407	966	Complete	Yes
SlituAPA	*Spodoptera litura*	Noctuidae	GenBank	XP_022814658	935	Complete	Yes
SlituPSA	*Spodoptera litura*	Noctuidae	GenBank	XP_022830253	949	Complete	Yes
SlituIRAP	*Spodoptera litura*	Noctuidae	GenBank	XP_022832232	1101	Complete	Yes
TnAPN1	*Trichoplusia ni*	Noctuidae	GenBank	AAX39863	982	Complete	Yes
TnAPN2	*Trichoplusia ni*	Noctuidae	GenBank	AAX39864	938	Complete	Yes
TnAPN3	*Trichoplusia ni*	Noctuidae	GenBank	AAX39865	940	Complete	Yes
TnAPN4	*Trichoplusia ni*	Noctuidae	GenBank	AAX39866	948	Complete	Yes
TnAPN5	*Trichoplusia ni*	Noctuidae	GenBank	AEA29693	940	Complete	Yes

TnAPN6	*Trichoplusia ni*	Noctuidae	GenBank	AEA29694	959	Complete	Yes

†The names of these lepidopteran M1 aminopeptidase genes have been adjusted to a uniform nomenclature.

‡Gene ID represents the gene accession number of the GenBank database (http://www.ncbi.nlm.nih.gov/), SilkDB (http://silkworm.genomics.org.cn/), MonarchBase (http://monarchbase.umassmed.edu/) or Manduca Base (http://agripestbase.org/manduca/).

§Whether or not these M1 aminopeptidase genes were used in the phylogenetic analysis in Fig. 1b.

Supplementary Table 3. Primary and secondary antibodies used in this study

Protein name	Catalog number	Supplier name	Clone name	Description	Dilution
APN1	—	Produced in this study	—	Rabbit polyclonal primary antibody	1:20000
APN3a	—	Produced in this study	—	Rabbit polyclonal primary antibody	1:20000
p38	ab170099	Abcam	E229	Rabbit monoclonal primary antibody	1:7500
JNK	ab179461	Abcam	EPR16797-211	Rabbit monoclonal primary antibody	1:5000
ERK	ab184699	Abcam	EPR17526	Rabbit monoclonal primary antibody	1:2000
p-p38	CST9215	Cell Signaling Technology	3D7	Rabbit monoclonal primary antibody	1:1250
p-JNK	ab4821	Abcam	—	Rabbit polyclonal primary antibody	1:5000
p-ERK	CST4370	Cell Signaling Technology	D13.14.4E	Rabbit monoclonal primary antibody	1:2000
β-actin	ab8227	Abcam	—	Rabbit polyclonal primary antibody	1:2000
—	CW0103	CWBIO	—	Goat anti-rabbit HRP-conjugated secondary antibody	1:5000

Supplementary Table 4. Mutagenesis of *PxAPN1* and *PxAPN3a* genes by CRISPR/Cas9

Genes	G0				G1		G2	
	Injected eggs	Hatched eggs (%)	Pupae (%)	Mutants (%)	Pupae	Heterozygotes (%)	Pupae (%)	Homozygotes (%)
PxAPN1	211	117 (55)	70 (60)	34 (49)	98	38 (39)	128	26 (20)
PxAPN3a	218	131 (60)	69 (53)	31 (45)	92	40 (43)	132	28 (21)

A mixture of Cas9 protein (300 ng/μl) and the gene-specific single guide RNA (sgRNA, 150 ng/μl) targeting either exon 12 of *PxAPN1* or exon 13 of *PxAPN3a* were microinjected into more than 200 fresh eggs of generation 0 (G0) (Supplementary Figs. 5a, e). Nondestructive genotyping of indel mutations around the sgRNA target site by direct sequencing of G0 individuals was used to demonstrate the site-specific mutagenic efficiency (45-50%) in G0 individuals of each group (Supplementary Figs. 5b, f). The resulting, mutated, G0 adults were sibling crossed to produce the progeny of next generation (G1), direct sequencing and further TA cloning and sequencing of G1 individuals revealed 7 and 10 different types of indel mutations for *PxAPN1* (Supplementary Fig. 5c) and *PxAPN3a* (Supplementary Fig. 5g), respectively, indicating that the mutated gene alleles in both groups could be transmitted to the next generation. For *PxAPN1* gene knockout, the G1 mutant heterozygotes contained 5 biallelic heterozygous mutants and 33 monoallelic mutants. Among them, the 33 monoallelic mutants contained 5 different mutation types: type 1 (n = 20, with 2-bp deletion), type 2 (n = 6, with 4-bp deletion), type 3 (n = 3, with 11-bp deletion), type 4 (n = 3, with 7-bp deletion and 3-bp insertion), type 5 (n = 1, with 10-bp deletion and 2-bp insertion) (Supplementary Fig. 5c). For *PxAPN3a* gene knockout, the G1 mutant heterozygotes contained 4 biallelic heterozygous mutants and 36 monoallelic mutants. Among them, the 36 monoallelic mutants contained 5 different mutation types: type A (n = 22, with 7-bp deletion), type B (n = 8, with 3-bp deletion), type C (n = 4, with 10-bp deletion), type D (n = 1, with 11-bp deletion and 3-bp insertion), type E (n = 1, with 14-bp deletion and 4-bp insertion) (Supplementary Fig. 5g). Adults with the most common indel mutation types for *PxAPN1* (n = 20, with 2-bp deletion) and *PxAPN3a* (n = 22, with 7-bp deletion) were further sib-crossed in each group to obtain the G2 strain. More than 100 G2 individuals in each group were nondestructively genotyped by direct sequencing, finally, the 26 homozygous mutants (12 males and 14 females) from the APN1KO strain were sib-crossed to create a stable homozygous mutant strain APN1KO in G3, and the 28 homozygous mutants (15 males and 13 females) from the APN3aKO strain were also sib-crossed to create another stable homozygous mutant strain APN3aKO in G3 (Supplementary Figs. 5d, h).

Supplementary Table 5. Experimental UPLC-MS/MS parameters used for JH and 20E detection by MRM in positive ion mode

Compound	Transitions (*m*/*z*)	CV (V)*	CE (V)†	DT (ms)‡	Proposed production§
JH II	281.2→249.1	4	6	54	$[M\text{–}CH_3OH+H]^+$
	281.2→147.0	4	12	54	$[M\text{–}COOCH_3\text{–}CH_2{=}CHCH_2CH_3\text{–}H_2O]^+$
	281.2→ 84.9	4	14	54	$[M\text{–}CH(CH_2)_2C(CH_3)CHCOOCH_3\text{–}CCH_2(CH_3)_2+H]^+$
JH III	267.2→235.2	22	6	110	$[M\text{–}CH_3OH+H]^+$
	267.2→147.1	22	10	110	$[M\text{–}COOCH_3\text{–}CH_2{=}CHCH_3\text{–}H_2O]^+$
	267.2→109.0	22	10	54	$[M\text{–}C(CH_3)_2OCH(CH_2)_2CH_3\text{–}CH_3OH]^+$
Methoprene	311.2→219.1	2	8	65	$[M\text{–}CH_3OH\text{–}CH(CH_3)_2OH+H]^+$
	311.2→191.1	2	10	65	$[M\text{–}CH_3OH\text{–}CH(CH_3)_2OH\text{–}CO+H]^+$
20E	481.1→445.2	28	12	81	$[M\text{–}2H_2O+H]^+$
	481.1→371.2	28	12	81	$[M\text{–}C(CH_3)_2CH_2OH\text{–}2H_2O]^+$
22S, 23S-homobrassinolide	495.3→127.0	44	16	81	$[M\text{–}C_{21}H_{33}O_4\text{–}H_2O]^+$
	495.3→109.0	44	28	81	$[M\text{–}C_{21}H_{33}O_4\text{–}2H_2O]^+$

*Core voltage (V).

†Collision energy (V).

‡Dwell time (ms) in the auto dwell mode.

§The structure of the proposed product $C_{21}H_{33}O_4$ is:

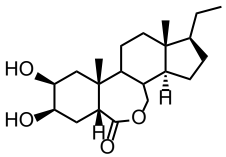

Supplementary Table 6. Primers used in this study

Purpose	Gene name	Primer name	Primer sequence (5´-3´)	PCR product (bp)	Tm (°C)	Positions (bp)[#]
Full-length cDNA cloning	PxAPN1[¶]	fAPN1-F	TATACTGGCGAGAAAATGG	2979	55	-15–2964
		fAPN1-R	CACAACAAAATTGATAGCG			
	PxAPN2	fAPN2-F	GTGCTGTTTTAGGATAATAAA	2953	55	-43–2910
		fAPN2-R	AGGCACCAAATGTTCAA			
	PxAPN3a	fAPN3a-F	TCCACCTATAGTTAGTGCC	3151	55	-67–3084
		fAPN3a-R	ATAAACCTTTTAGACCAGAAT			
	PxAPN4a	fAPN4a-F	TCATTTATTGGATTACGGC	2952	55	-26–2926
		fAPN4a-R	TATTTAGGCACTCAAAGACT			
	PxAPN4b	fAPN4b-F	CACCGAATAGTAGCGAT	2951	51	-90–2861
		fAPN4b-R	CAAAACATAGTTCAGTAAAA			
	PxAPN5	fAPN5-F	ATTTAAGGCTAAGCAAAGTG	2988	55	-152–2836
		fAPN5-R	CAAATAAGCTGGCAATCAG			
	PxAPN6	fAPN6-F	AATCCATTTTATTAGGTAGG	2967	51	-56–2911
		fAPN6-R	TTTATTGATCGACGACAA			
	PxAPN7	fAPN7-F	AATTCAGCCAGTGCGTAT	2629	57	-28–2601
		fAPN7-R	CGTTTGTAAGGCATTAGTATG			
	PxAPN8	fAPN8-F	CTGGGACTGGAGGTAGACA	2788	60	-18–2760
		fAPN8-R	CAGGATCACCAGCAACGA			
	PxAPN9	fAPN9-F	ATGACTCGTGCCAAGTAG	3084	57	-53–3031
		fAPN9-R	CAAACTAAACCCTCTGCTA			
	PxAPN10[¶]	fAPN10-F	ATCAAGACACGGAGCATAGA	2816	60	85–2900
		fAPN10-R	AAAAGACAATACTGGCATTACAC			
	PxAPN11-1	fAPN11-1-F	GAGTCTCAAGCCGCAGAG	3525	60	40–3564

		fAPN11-1-R	AAATCCGTAACGAACAACG			
	PxAPN11-2	fAPN11-2-F	AAGCCAATTTACAATGTCC	3455	60	-13–3442
		fAPN11-2-R	ATCCGTAACGAACAACGCT			
	PxAPN12	fAPN12-F	GCCTGGAGTGAAAACCTG	3099	60	-77–3022
		fAPN12-R	GCTATCCTTGTCGCTCTTT			
	PxAPN13¶	fAPN13-F	ATCTTCATCTAACCCTCATTCC	3119	60	6–3124
		fAPN13-R	AAAACCCTTGGTCTACAACAGT			
	PxAPA-1	fAPA1-F	TGGCAAATGGCAATACTAAA	3144	60	-172–2972
		fAPA1-R	TTCACAGGGCAAACAATCTA			
	PxAPA-2	fAPA2-F	TGGTTTGTTTGTTACGGAT	2847	60	29–2875
		fAPA2-R	TTCTGCTAAACGACTGGAC			
	PxPSA¶	fPSA-F	CGGTCCGAACATTTCTTT	2966	60	-110–2856
		fPSA-R	TGTGGCTCAGTGGTAGGC			
	PxIRAP¶	fIRAP-F	ATAAGGCGGACAGGAACG	3286	60	35–3320
		fIRAP-R	CTCTAAACCCCGGCATACTC			
gDNA fragment cloning	PxAPN6	gAPN6-F	GTTCTTGAAACCATGGAGCACTC	1870	55	Exon 12–16
		gAPN6-R	CGACGACAATGTCAACGTAAC			
qPCR analysis	PxAPN1	qAPN1-F	TCACTGAGTCCCATCCCA	157	55	58–214
		qAPN1-R	TGCCAGACGGCACATTTT			
	PxAPN2	qAPN2-F	TTCCCGTATGCCTTCCC	127	55	868–994
		qAPN2-R	TCGTCGTCACGCCTTCT			
	PxAPN3a	qAPN3a-F	GGCTACCGTTGGCTACAC	110	50	2190–2299
		qAPN3a-R	GCAGACATTCCTCCACTCC			
	PxAPN4a	qAPN4a-F	CATCCGTATTCACGACAAA	169	55	2466–2643
		qAPN4a-R	CAGGTATGCAGCAAGAGTG			
	PxAPN4b	qAPN4b-F	TGGAGCATTTAGTCACGC	147	55	1346–1492

	qAPN4b-R	CAGGCAACTGGCTTAGAG			
PxAPN5	qAPN5-F	GGACGATCAGGCTGTTAA	160	55	2412–2571
	qAPN5-R	TTCCCGAATCTGGTTGTG			
PxAPN6	qAPN6-F	CAGCAGGGACGGATAGAA	146	53	65–210
	qAPN6-R	ATCGGTAGCAACGAAGTTAA			
PxAPN7	qAPN7-F	CATCACCTTCGCTTGCTC	141	55	2151–2291
	qAPN7-R	TATCCGTCGCTGTGGTTG			
PxAPN8	qAPN8-F	GCCGTCTTTGTTCTCACT	106	55	538–643
	qAPN8-R	CATTCCTGCTGTATCCATT			
PxAPN9	qAPN9-F	TTTCGCATAACTTACAGCG	107	55	568–674
	qAPN9-R	GCATTACTTGGGTGCAGAT			
PxAPN10	qAPN10-F	TTACCAGCCCTGCGTGAC	145	55	2154–2298
	qAPN10-R	CAGACCAAACTCCCATTCCT			
PxAPN11	qAPN11-F	CAGAACTCCACCGACCCT	131	55	2704–2834
	qAPN11-R	GCGTACACGGCGTATTGC			
PxAPN12	qAPN12-F	ATTGTGGACGACGCTATGA	165	58	2089–2253
	qAPN12-R	ATAATGCGCTCCTTTCGA			
PxAPN13	qAPN13-F	TAAGGTGGATCTGAATGACACG	116	55	384–499
	qAPN13-R	GCTCGCTCTTCAAGTTACCG			
PxAPA	qAPA-F	GTGGTTTGGGAATCTGGT	106	58	1101–1206
	qAPA-R	GGATGGCTCAATAGCGTCTA			
PxPSA	qPSA-F	CTTGGACTAAGCAGATGGGATT	154	55	1604–1757
	qPSA-R	TGCGTGGAGATTGTGATGG			
PxIRAP	qIRAP-F	AAACACTTCGTATCCACTGCCTAA	161	55	1287–1447
	qIRAP-R	AAACCAGACCGGAGAACCATT			
PxmALP	qALP-F1	GCACACACCATGACCGTAGCAG	169	61	1207–1375

		qALP-R1	GGCTCTTCGTGACATCG			
	PxABCB1	qABCB1-F	AGCGAAAGGAGATTGATAGG	157	55	942–1098
		qABCB1-R	GTAATAAACTGGAAACCGAAC			
	PxABCC1	qABCC1-F	GGTGGTGCTCATCTGCTACCTCAT	165	55	696–860
		qABCC1-R	ATCCTGACACGCTCATCGGTTTT			
	PxABCC2	qABCC2-F	AGTCTTGGCACGCAAACGG	103	53	2401–2503
		qABCC2-R	CGAACAGACGCATGAAGGACAT			
	PxABCC3	qABCC3-F	TCAACCGCTTCACCAAGGACAT	111	55	2606–2716
		qABCC3-R	CGGCGTTCAGCACCAGGAT			
	PxABCG1	qABCG1-F	ATCTGGTGTTTCAGGCTTTAGTC	118	60	545–662
		qABCG1-R	ATCACGGTGTTCTGGCATTT			
	PxMAP4K4	qMAP4K4-F	CATCAACTGGCTCCGTCTG	188	63	7–215
		qMAP4K4-R	TCATCTTCGGTGACATCCATC			
	PxRPL32	qL32-F	CCAATTTACCGCCCTACC	120	50–60	—
		qL32-R	TACCCTGTTGTCAATACCTCT			
Heterologous expression*	PxAPN1	APN1-Exp-F	CTCAAGCTTCGAATT***GCCACC***ATGGATTCTCGCTGGTTCC	2967	60	1–2967
		APN1-Exp-R	GGCGACCGGTGGATC***TCCTCCTCCTCC***CACAACAAAATTGATAGCGACG			
	PxAPN3a	APN3a-Exp-F	CTCAAGCTTCGAATT***GCCACC***ATGGCGACAACGACAAC	3075	60	1–3075
		APN3a-Exp-R	GGCGACCGGTGGATC***TCCTCCTCCTCC***GACCAGAATATTTGCGATCAC			
	PxAPN5	APN5-Exp-F	CTCAAGCTTCGAATT***GCCACC***ATGGCTCTTCTTCTGAAGTT	2853	60	1–2853
		APN5-Exp-R	GGCGACCGGTGGATC***TCCTCCTCCTCC***C			

			TTCATCAATAGCGCAAATAAGCTG			
	PxAPN6	APN6-Exp-F	CTCAAGCTTCGAATT***GCCACC***ATGTTCCGGGTAACAACTGTTG	2880	60	1–2880
		APN6-Exp-R	GGCGACCGGTGGATC***TCCTCCTCCTCC***AACTATCAAAGTAATCATTGC			
dsRNA synthesis[†]	PxAPN1	dsAPN1-F	T7-TGACGCTAACACCTTACTTTG	382	55	176–511
		dsAPN1-R	T7-AGCTGCGATAGAACCCTCT			
	PxAPN3a	dsAPN3a-F	T7-AGCAGCCTTCGCTCTTCC	357	55	48–358
		dsAPN3a-R	T7-CGATGTTGAGGTCCTTTCC			
	PxAPN5	dsAPN5-F	T7-GGCTCTTCTTCTGAAGTTGG	403	55	3–359
		dsAPN5-R	T7-AAGGGTGATGATGGGTTTA			
	PxAPN6	dsAPN6-F	T7-TTTAAGCATTTTGCTCTTTG	378	55	24–355
		dsAPN6-R	T7-ATGGCATTGGTTTTCCTG			
	PxMAP4K4	dsMAP4K4-F	T7-GCCCGAGATACGCAAATACA	582	59	2739–3274
		dsMAP4K4-R	T7-CCGAGCCATAGATCACTTTCA			
	EGFP	dsEGFP-F	T7-CCACAAGTTCAGCGTGTCCG	469	60	—
		dsEGFP-R	T7-AAGTTCACCTTGATGCCGTTC			
CRISPR sgRNA[‡]	PxAPN1	CRISPR-N1-F	*GAAATTAATACGACTCACTATAGGG***AGGCCGTGGGTGTACTGCGC**GTTTTAGAGCTAGAAATAGC	125	70	Exon 12
		CRISPR-R	AAAAGCACCGACTCGGTGCCACTTTTTCAAGTTGATAACGGACTAGCCTTATTTTAACTTGCTATTTCTAGCTCTAAAAC			
	PxAPN3a	CRISPR-N3a-F	*GAAATTAATACGACTCACTATAG***GGCGTAGCGATGCCGTCTCG**GTTTTAGAGCTAGAAATAGC	123	70	Exon 13

		CRISPR-R	AAAAGCACCGACTCGGTGCCACTTTTTC AAGTTGATAACGGACTAGCCTTATTTTAA CTTGCTATTTCTAGCTCTAAAAC			
Specific sequencing primers[§]	PxAPN1	APN1-F	GCCTGTGTCTTTCAGTGGACTGTAC	297	55	—
		APN1-R	GCTCGTTGTCTTCTACTACACTATTGAG			
	PxAPN3a	APN3a-F	AGCACGGGTCTCCATCCAATAA	294	55	—
		APN3a-R	CGGTCGAAAATTTACGGACTG			

*Restriction enzymes *Eco*RI and *Bam*HI were used to cut the pie2-GFP-N1 expression vector and generate a linearized vector, PCR primers used to amplify the genes for expression (PxAPN1-Exp, PxAPN3a-Exp, PxAPN5-Exp, PxAPN6-Exp) contained 15 bp extensions (underlined) homologous to the cut vector ends. Before the ATG initiation codon Kozak consensus sequences were included (italicized and emboldened) to increase the translation efficiency. In addition, there are linker sequences encoding four tryptophans (italicized and emboldened) in PxAPN1-Exp-R, PxAPN3a-Exp-R, PxAPN5-Exp-R, PxAPN6-Exp-R. Genes cloned into the multiple cloning site (MCS) were expressed as PxAPN-GFP fusion proteins.

[†]Forward and reverse primers used to synthesize specific dsRNA templates contained the T7 RNA polymerase promoter sequence (5′-TAATACGACTCACTATAGGGAGA-3′) appended to both their 5′- and 3′-ends.

[‡]Specific oligonucleotides encoding T7 polymerase-binding sites (italicized) and the sgRNA target sequences (underlined and bold) of the *PxAPN1* and *PxAPN3a* genes were respectively designed as the forward primers CRISPR-N1-F, CRISPR-N3a-F, and a common oligonucleotide encoding the remaining sequences of CRISPR-N1-F, CRISPR-N3a-F were designed as the reverse primer CRISPR-R.

[§]To further identify the precise indel mutation types, the gDNA fragments (297 bp and 294 bp) of *PxAPN1* and *PxAPN3a* genes flanking the CRISPR target site were amplified and the PCR products of the mutants were directly sequenced or ligated into *pEASY*-T1 cloning vector and sequenced for further validation.

[¶]Owing to the high GC contents of these genes, high GC buffer with LA Taq polymerase was used to clone their full-length cDNA sequence.

[#]Positions indicating the full-length cDNA coding regions of all these genes deposited in GenBank (accession nos. MG873047–MG873063 and MH213067–MH213068).

Supplementary References

1. Xie, W. *et al.* Tissue-specific transcriptome profiling of *Plutella xylostella* third instar larval midgut. *Int. J. Biol. Sci.* **8**, 1142–1155 (2012).
2. Lei, Y. *et al.* Midgut transcriptome response to a Cry toxin in the diamondback moth, *Plutella xylostella* (Lepidoptera: Plutellidae). *Gene* **533**, 180–187 (2014).
