# Supplementary material for: MAPK-dependent hormonal signaling plasticity contributes to overcoming Bacillus thuringiensis toxin action in an insect host
Source: Nat Commun. 2020 Jun 12;11:3003. doi: 10.1038/s41467-020-16608-8 (PMC7293236; doi:10.1038/s41467-020-16608-8)
Supplement: Supplementary file 3 — Reporting Summary [file 41467_2020_16608_MOESM3_ESM.pdf]

nature research

Corresponding author(s): Zhaojiang Guo, Youjun Zhang

Last updated by author(s): Apr 5, 2020

Reporting Summary

Nature Research wishes to improve the reproducibility of the work that we publish. This form provides structure for consistency and transparency in reporting. For further information on Nature Research policies, see Authors & Referees and the Editorial Policy Checklist .

Statistics

For all statistical analyses, confirm that the following items are present in the figure legend, table legend, main text, or Methods section.

n/a	Confirmed	
☐	☒	The exact sample size (n) for each experimental group/condition, given as a discrete number and unit of measurement
☐	☒	A statement on whether measurements were taken from distinct samples or whether the same sample was measured repeatedly
☐	☒	The statistical test(s) used AND whether they are one- or two-sided<br>*Only common tests should be described solely by name; describe more complex techniques in the Methods section.*
☒	☐	A description of all covariates tested
☒	☐	A description of any assumptions or corrections, such as tests of normality and adjustment for multiple comparisons
☐	☒	A full description of the statistical parameters including central tendency (e.g. means) or other basic estimates (e.g. regression coefficient) AND variation (e.g. standard deviation) or associated estimates of uncertainty (e.g. confidence intervals)
☒	☐	For null hypothesis testing, the test statistic (e.g. F, t, r) with confidence intervals, effect sizes, degrees of freedom and P value noted<br>*Give P values as exact values whenever suitable.*
☒	☐	For Bayesian analysis, information on the choice of priors and Markov chain Monte Carlo settings
☒	☐	For hierarchical and complex designs, identification of the appropriate level for tests and full reporting of outcomes
☒	☐	Estimates of effect sizes (e.g. Cohen's d, Pearson's r), indicating how they were calculated

Our web collection on statistics for biologists contains articles on many of the points above.

Software and code

Policy information about availability of computer code

Data collection	qPCR: QuantStudio 3; Western blot: Tanon-5200; confocal: ZEN 2012; hormone detection: MassLynx 4.1
Data analysis	Gene sequence analysis: DNAMAN 9.0; gene primer design: Primer Premier 5.0; protein sequence analysis: Translate, Compute pI/Mw, SignalP 4.1, big-PI Predictor, GPI-SOM, TMHMM 2.0, NetNGlyc 1.0, NetOGlyc 4.0 and WebLogo 3; phylogenetic analysis: MEGA 7.0; Western blot: ImageJ 1.51; statistic analysis: SPSS 23.0; figure drawing: Microsoft Office 2010, SigmaPlot 14.0 and GraphPad Prism 8.3; chemical structure: ChemDraw 2014

For manuscripts utilizing custom algorithms or software that are central to the research but not yet described in published literature, software must be made available to editors/reviewers. We strongly encourage code deposition in a community repository (e.g. GitHub). See the Nature Research guidelines for submitting code & software for further information.

Data

Policy information about availability of data

All manuscripts must include a data availability statement. This statement should provide the following information, where applicable:

- Accession codes, unique identifiers, or web links for publicly available datasets
- A list of figures that have associated raw data
- A description of any restrictions on data availability

The full-length cDNA sequences of all the cloned genes in this study have been deposited in the GenBank database (accession nos. MG873047–MG873063 and MH213067–MH213068). The authors declare that all the data supporting the findings in this study are available in the manuscript and its Supplementary Information. The source data underlying Figs. 1c&d, 2b-f, 3, 4, 5b-f, 6, 7b-i&k-r, 8 and Supplementary Figs. 1a, 2, 3a&b, 4, 6 and 8 are provided as a Source Data file.

Field-specific reporting

Please select the one below that is the best fit for your research. If you are not sure, read the appropriate sections before making your selection.

☒ Life sciences ☐ Behavioural & social sciences ☐ Ecological, evolutionary & environmental sciences

For a reference copy of the document with all sections, see nature.com/documents/nr-reporting-summary-flat.pdf

Life sciences study design

All studies must disclose on these points even when the disclosure is negative.

Sample size	Sample-size calculations were not required for all experiments of this study, sample sizes were selected based on our previous experience to obtain statistical significance and reproducibility (Guo et al., 2015, PLoS Genetics, 11(4): e1005124). All samples sizes are indicated in figure legends or the corresponding methods.
Data exclusions	No data were excluded from the analyses.
Replication	Experimental findings were reliably reproduced in at least three independent experiments as indicated throughout the manuscript.
Randomization	All insect individuals in our experiments were randomly allocated into different experimental groups.
Blinding	Blinding was not relevant to molecular experiments conducted in this study because all cells/samples were analyzed in the same way.

Reporting for specific materials, systems and methods

We require information from authors about some types of materials, experimental systems and methods used in many studies. Here, indicate whether each material, system or method listed is relevant to your study. If you are not sure if a list item applies to your research, read the appropriate section before selecting a response.

Materials & experimental systems

n/a	Involved in the study	
☐	☒	Antibodies
☐	☒	Eukaryotic cell lines
☒	☐	Palaeontology
☐	☒	Animals and other organisms
☒	☐	Human research participants
☒	☐	Clinical data

Methods

n/a	Involved in the study	
☒	☐	ChIP-seq
☒	☐	Flow cytometry
☒	☐	MRI-based neuroimaging

Antibodies

Antibodies used	All the primary and secondary antibodies used in this study have been described in detail in the Supplementary Table 3. Anti-APN1/3a (produced in this study, 1:20000); anti-p38 (Abcam, #ab170099, clone E229, 1:7500); anti-JNK (Abcam, #ab179461, clone EPR16797-211, 1:5000); anti-ERK (Abcam, #ab184699, clone EPR17526, 1:2000); anti-p-p38 (Cell Signaling Technology, #CST9215, clone 3D7, 1:1250); anti-p-JNK (Abcam, #ab4821, 1:5000); anti-p-ERK (Cell Signaling Technology, #CST4370, clone D13.14.4E, 1:2000); anti-β-actin (Abcam, #ab8227, 1:2000).
Validation	The validation of antibodies prepared in our laboratory has been provided in this study. The validation statements of commercial antibodies are available on the respective manufacturer's website. Anti-p38 (species: mouse, rat, human, etc.; application: WB, ICC/IF, IP, Flow Cyt, ChIP); anti-JNK (species: mouse, rat, cow, dog, human, monkey, zebrafish, Xenopus tropicalis, etc.; application: WB, ICC/IF, IP, Flow Cyt); anti-ERK (species: mouse, rat, human, etc.; application: WB, ICC/IF, IP, Flow Cyt); anti-p-p38 (species: human, mouse, rat, hamster, monkey, mink, chicken, Drosophila melanogaster, Xenopus, zebrafish, bovine, dog, pig, Saccharomyces cerevisiae, Caenorhabditis elegans, horse, etc.; application: WB, IHC, IF, IP, Flow Cyt, ChIP, ELISA); anti-p-JNK (species: mouse, human, etc.; application: WB, ICC/IF, IHC-P, IHC-Fr); anti-p-ERK (species: human, mouse, rat, hamster, monkey, mink, chicken, Drosophila melanogaster, Xenopus, zebrafish, bovine, dog, pig, Saccharomyces cerevisiae, Caenorhabditis elegans, horse, etc.; application: WB, IHC, IF, IP, Flow Cyt, ChIP, ELISA); anti-β-actin (species: not confirmed experimentally; application: IHC-Fr, IP, WB, ICC, Flow Cyt, IHC-FrFl, IHC-P, IHC-P, ICC/IF, ELISA).

Eukaryotic cell lines

Policy information about cell lines

Cell line source(s)	The insect cell line Sf9 was purchased from Invitrogen.

Authentication	The insect cell line Sf9 was obtained from original source and was not further authenticated.
Mycoplasma contamination	The insect cell line Sf9 was tested negative for mycoplasma contamination.
Commonly misidentified lines (See ICLAC register)	No commonly misidentified cell line was used.

Animals and other organisms

Policy information about studies involving animals; ARRIVE guidelines recommended for reporting animal research

Laboratory animals	Diamondback moth, Plutella xylostella.<br>The five different P. xylostella strains used in this study have been described in detail in the Methods section of the manuscript and our previous studies (Guo et al., 2015, PLoS Genetics, 11(4): e1005124; Guo et al., 2015, Journal of Invertebrate Pathology, 126, 21–30; Zhu et al., 2015, Pest Management Science, 71(2): 225–233). The DBM1Ac-S strain is susceptible to Btk formulation and Bt toxins, and the Bt-resistant strains including DBM1Ac-R, NIL-R and SZ-R have developed approximately 3500-, 4000-, and 450-fold resistance to Cry1Ac protoxin compared to the susceptible DBM1Ac-S strain, while SH-R larvae have developed about 1900-fold resistance to Btk formulation compared to the DBM1Ac-S strain. Male and female P. xylostella samples from different developmental stages were used in different experiments.
Wild animals	The study did not involve wild animals.
Field-collected samples	The study did not involve samples collected from the field.
Ethics oversight	No ethical oversight was required as no vertebrate animals were involved in the study, and no ethical oversight of the experiments was required by our institution.

Note that full information on the approval of the study protocol must also be provided in the manuscript.
